# Hemodynamics, plasma catecholamines, and power spectral analysis of heart rate variability during tilt-evoked neurally mediated hypotension

**DOI:** 10.3389/fnins.2026.1929006

**Published:** 2026-10-06

**Authors:** Guillaume Lamotte, Patti Sullivan, David S. Goldstein

**Affiliations:** 1 Clinical Neurosciences Program/Division of Intramural Research (DIR)/National Institute of Neurological Disorders and Stroke (NINDS)/National Institutes of Health (NIH), Bethesda, MD, United States; 2 Department of Neurology, University of Utah, Salt Lake City, UT, United States; 3 George E. Wahlen VA Medical Center, Salt Lake City, UT, United States

**Keywords:** baroreflex, hypotension, sympathetic, sympathoadrenal, syncope

## Abstract

**Background:**

Syncope workup often includes tilt table testing. One positive result is neurally mediated hypotension (NMH). Mechanisms of NMH are debated.

**Objectives:**

We characterized NMH development in terms of systemic and forearm hemodynamics, plasma catecholamines, and power spectral analysis of heart rate variability.

**Methods:**

We reviewed data from 25 subjects with NMH and 34 controls without NMH who had autonomic testing between 2003 and 2024. Dependent measures included continuously recorded blood pressure (BP), heart rate, stroke volume, cardiac output, respiratory rate (RR), forearm blood flow (FBF), forearm vascular resistance (FVR), systemic vascular resistance (SVR), plasma epinephrine and norepinephrine, and high- and low-frequency power of heart rate variability (HF_RR_ and LF_RR_). There were 4 study periods: T0 = supine rest; T1 = start of tilt; T2 = NMH (20 min of tilting in controls); and T3 = supine recovery.

**Results:**

There were significant interaction effects for Group X Study Period for several variables, with a decrease in BP, FVR, SVR, HF_RR_, and LF_RR_, and an increase in FBF and epinephrine at a steeper rate in the NMH group compared to the No NMH group. The two groups did not differ in responses of heart rate, RR, stroke volume, cardiac output, or norepinephrine levels. The increment in plasma epinephrine was larger in the NMH group, without a group difference in norepinephrine (i.e., sympathoadrenal imbalance). LF_RR_ was elevated at baseline and decreased markedly in participants developing NMH.

**Conclusions:**

NMH is associated with forearm vasodilation, sympathoadrenal imbalance, and a precipitous fall in LF_RR_. The results fit with a neurocirculatory-neuroendocrine pattern triggering NMH.

## Introduction

Tilt table testing is often done to evaluate patients with repeated episodes of syncope or presyncope. A positive outcome is an acute fall in blood pressure before or concurrent with presyncope. Terms such as neurally mediated syncope, vasodepressor syncope, neurocardiogenic syncope, and vasovagal syncope have been used to describe this phenomenon. The mechanisms remain debated. We used the descriptive term tilt-evoked neurally mediated hypotension (NMH) because it does not overemphasize a particular trigger or pathophysiological mechanism.

We performed provocative tilt table testing and a comprehensive evaluation of hemodynamic and neurohumoral responses before, during, and after NMH. The objective was to identify the physiological triggers and systemic response patterns that lead to NMH. We hypothesized that NMH would be triggered by a centrally evoked neuroendocrine pattern that includes sympathoadrenal imbalance (i.e., a disproportionately large increase in epinephrine compared to norepinephrine Goldstein et al., 2003), resulting in acute decreases in skeletal vasomotor tone and shunting cardiac output away from the brain, leading to presyncope.

## Methods

### Subjects

The Institutional Review Board of the NINDS approved the study (data analyzed in this study were drawn from tabulated results and databases for the following NIH intramural clinical research protocols: 03N0004, 03N0140, 16N0058, 000089, 18N0140). The original protocols' informed consent included secondary analysis and the public sharing of anonymized individual data, as detailed in the Supplementary material. A separate ethics review was not necessary for this pooled secondary analysis of de-identified data per the NIH Data Management and Sharing Policies. We reviewed results from all the provocative tilt table tests done at the National Institutes of Health Clinical Center between 2003 and 2024. This study focused solely on tests using the 40-min tilt table test protocol, chosen to detect delayed responses, presyncope, or other orthostatic conditions distinct from neurogenic orthostatic hypotension.

We identified NMH by an acute decrease in blood pressure associated with presyncope (Supplementary Figure S1), characterized by pallor, lightheadedness, nausea, diaphoresis, visual scotomata, vision loss, or impending loss of consciousness with hypotension. Hypotension and presyncope in patients with NMH resolved within 30 s after returning to the supine position. None of the NMH patients lost consciousness during the testing. We differentiated NMH from classic and delayed orthostatic hypotension by normal blood pressure responses to the Valsalva maneuver in all participants included (i.e., preservation of late Phase II blood pressure, presence of a Phase IV blood pressure overshoot, and normal pressure recovery time), which was not consistent with neurogenic orthostatic hypotension (Jones and Gibbons, 2014).

### Instrumentation and head-up tilt table testing

Non-invasive monitoring devices included a brachial automated cuff and mercury in silastic strain gauge for measurement of forearm blood flow (FBF) by impedance plethysmography (DE Hokanson, Inc., Bellevue, WA), metal plates on two fingers for tracking skin electrical conductance (SEC, ADInstruments, Colorado Springs, CO), and a finger cuff for continuous blood pressure (BP) tracking [Finapres Nova (Finapres Medical Systems, Enschede, The Netherlands); or Nexfin (BMEYE, Amsterdam, The Netherlands)]. We calculated cardiac stroke volume (SV) using software bundled with the finger cuff systems. Cardiac output (CO) was calculated from the SV times the simultaneously measured heart rate (HR). Forearm vascular resistance (FVR) was calculated from the mean arterial pressure (MAP) divided by the FBF, and systemic vascular resistance (SVR) from the MAP (−6 mmHg for assumed central venous pressure) divided by the CO.

All study participants underwent intravenous (IV) blood collection. We drew blood samples for plasma norepinephrine (NE) and epinephrine (EPI) through an indwelling IV placed at the beginning of the study to avoid pain from venous puncture during tilt, which could trigger reflex syncope. With the subject supine, we inserted an intravenous catheter into an arm vein and connected it via a 3-way stopcock to a slow-rate normal saline infusion to keep the vein open.

Before tilting and after resting supine for at least 10 min, we obtained baseline heart rate variability data. We assessed time- and frequency-domain heart rate variability using the HRV module bundled with the PowerLab recording device (LabChart 8.1.13, ADInstruments, Colorado Springs, CO).

Each subject performed a Valsalva maneuver while supine (30 mmHg, 12 s) at least three times until a technically adequate tracing was obtained. Baroreflex-cardiovagal function was quantified from the slope of the linear relationship between cardiac interbeat interval (in seconds) and the systolic blood pressure (in mmHg) during Phase II of the Valsalva maneuver, as described previously (Goldstein et al., 1982). To evaluate baroreflex-sympathoneural function, we used the baroreflex areas method for Phases II and III/IV of the Valsalva maneuver (Rahman and Goldstein, 2014).

For head-up tilt table testing, the motorized table was brought to 70 degrees from horizontal over about 5–10 s. Beginning at about 2.5 min of tilting, FBF was measured, and at 4 min a blood sample was drawn through the IV. At least two investigators monitored the patient and physiological variables continuously: one viewed the signals, and one monitored the patient. FBF and blood sampling were repeated every 4 min for up to 40 min and after 10 min of supine recovery. There was no attempt to provoke hypotension with a drug. No subject lost consciousness.

We summarize further details of head-up tilt table testing and instrumentation in the Supplementary material.

### Catechol assay

We assayed plasma NE and EPI by batch alumina extraction followed by liquid chromatography with series electrochemical detection (Holmes et al., 1994).

### Study periods

We defined four study periods: T0 = supine rest during the last 5 min in the supine position before head-up tilt; T1 = start of the tilt after the second minute in the 70° head-up tilt position (the first minute after head-up tilt was excluded to avoid transition phenomena); T2 = NMH including the last 3 min just before the end of tilting, or else 20 min of head-up tilt in subjects without NMH (No NMH); and T3 = supine recovery after 10 min of supine position following the end of tilting. Valsalva maneuvers, baseline catechol sampling, and six baseline FBF measurements were performed during the T0 window. We also performed baseline HRV analysis during the T0 window, before the Valsalva maneuvers, blood sampling, and FBF measurements. We separated the last Valsalva maneuver and the T0 variable measurements by at least 2 min to minimize possible interaction.

### Statistics

We performed statistical analyses using GraphPad Prism 10.1.0 (GraphPad Software, San Diego, CA). Continuous variables were expressed as means ± SEM. We used a linear mixed model as the primary analysis for comparisons between time points and groups (Supplementary Table S4) reports the effects of study periods (T0, T1, T2, T3) and groups (NMH vs. no NMH) on values for hemodynamic and neurochemical parameters using an analysis of variance (ANOVA) or mixed model effect. As a secondary analysis, we also compare mean absolute values in the NMH and No NMH groups at each of the four testing points using an independent-samples t-test. Frequencies of binary independent variables were compared between groups by Fisher's exact test. A p-value less than 0.05 defined statistical significance. Missing data were minimal for each variable. We are including the entire anonymized data spreadsheet as a Supplementary material.

## Results

Twenty-five participants had NMH; 34 did not. The NMH and No NMH groups were similar in age, gender, body mass, and medications (Supplementary Table S1). The NMH group more often reported a history of syncope and orthostatic intolerance (Supplementary Table S1).

All hemodynamic and neurochemical measures varied as a function of Study Period (Figure 1, Supplementary Table S4). Between-group comparisons demonstrated that the NMH and No NMH groups differed in hemodynamic responses during the tilting (Figure 1, Supplementary Tables S2–S4). There were significant interaction effects for Group X Study Period for several variables with a decrease in BP, FVR, SVR, HF_RR_, and LF_RR_, and an increase in FBF and EPI at a significantly steeper rate in the NMH group compared to the No MNH group (Supplementary Table S4). The two groups did not differ in responses of HR, respiratory rate, SV, CO, or NE levels (Supplementary Table S4).

**Figure 1:**
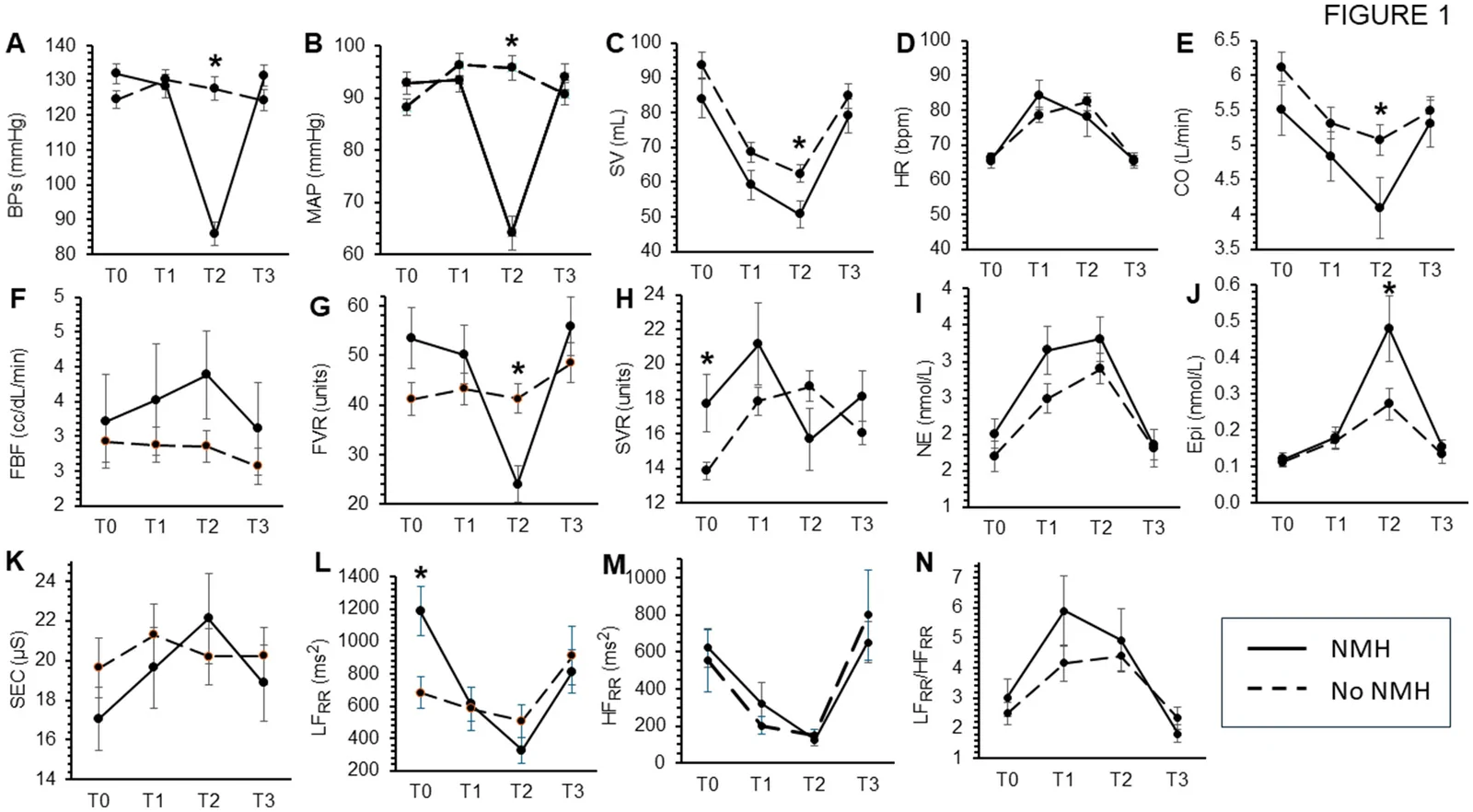
Hemodynamics and plasma catecholamines during tilt-evoked neurally mediated hypotension (NMH). Shown are mean (±SEM) values for systolic blood pressure (BPs) **(A)**, mean arterial pressure (MAP) **(B)**, stroke volume (SV) **(C)**, heart rate (HR) **(D)**, cardiac output (CO) **(E)**, forearm blood flow (FBF) **(F)**, forearm vascular resistance (FVR) **(G)**, and systemic vascular resistance (SVR) **(H)**, plasma norepinephrine (NE) **(I)**, plasma epinephrine (EPI) **(J)**, skin electrical conductance (SEC) **(K)**, low frequency power of heart rate variability (LF_RR_) **(L)**, high frequency power of heart rate variability (HF_RR_) **(M)**, and LF_RR_/HF_RR_ **(N)** during supine rest (T0), at the start of the head-up tilt test (T1), during tilt-evoked neurally mediated hypotension (NMH) or after 20 min of asymptomatic head-up tilt position for controls (T2), and during supine recovery (T3). *p < 0.05 between groups.

Between-group comparisons showed no difference in plasma EPI between the NMH and No NMH groups at T0 or T1. EPI increased from T0 to T1 in both groups; at T2, mean plasma EPI was higher in the NMH than in the No NMH group (p = 0.01).

The groups did not differ in mean SEC at any time point (Figure 1). In the NMH group, SEC increased between T1 and T2 (p < 0.001).

Baroreflex-cardiovagal and baroreflex-sympathoneural functions quantified using the baroreflex areas approach (Rahman and Goldstein, 2014) did not differentiate the groups (Supplementary Table S5). During NMH, we noted an absence of HR increase despite a rapid fall in BP (Figure 1).

At T0, mean LF_RR_ was higher in the NMH than in the No NMH group (p = 0.005; Figure 1, Supplementary Table S3). In the NMH group, there was a decrease in LF_RR_ from T0 to T1 (p < 0.001) and from T1 to T2 (p = 0.002), with no changes in the No NMH group (Figure 1, Supplementary Figure S2). In both the NMH and No NMH groups, HF_RR_ decreased between T0 and T1 (p = 0.004, p = 0.04; Figure 1). The ratio of LF_RR_/HF_RR_ did not differ as a function of Group in any study period (Figure 1).

## Discussion

We report that tilt-evoked NMH is associated with a particular pattern of acute neurocirculatory and neuroendocrine changes. Previous studies reported one or more aspects, but not this pattern revealed by comprehensive testing in the same patients. Our data best fit a centrally evoked pattern. We observed a constellation of forearm vasodilation, sympathoadrenal imbalance with disproportionate epinephrine release, acute baroreflex-cardiovagal withdrawal (evidenced by a rapid, precipitous fall in blood pressure without concurrent reflexive tachycardia and by a fall in LF_RR_) during development of NMH. Such a pattern cannot be accounted for by reflexive vagal activation and generalized sympathetic withdrawal (“vasovagal” reaction).

NMH was associated with prominent forearm vasodilation, in agreement with other studies (Barcroft and Edholm, 1945; Wieling et al., 2016). Studies using non-invasive continuous finger cuff measurements found reduced CO during NMH (Wieling et al., 2016). When CO has been measured invasively by thermodilution in patients with NMH, the proportionate decrease in MAP has been greater than that in CO, implying decreased SVR (Jardine et al., 2002).

Heart rate changes seem to contribute little to the NMH phenomenon. In other studies, in most individuals, HR has been reported to decrease at the time of syncope but only *after* a fall in BP (Jardine et al., 2002, 2018), so the role of bradycardia *per se* as a trigger in NMH is questionable, despite widespread use of the term “vasovagal” syncope.

The lack of HR increase despite a fall in BP implies withdrawal of baroreflex-cardiovagal function, as others have noted (Jardine et al., 2018). Whether baroreflex-sympathoneural function withdraws during NMH has been less clear. Tilt-evoked hypotension and syncope have been associated with a sudden cessation of muscle sympathetic nerve activity (MSNA) (Mosqueda-Garcia et al., 1997). Although some studies have not found a decrease in MSNA (Furlan et al., 2019), failure to increase MSNA substantially during hypotension suggests attenuated baroreflex-sympathoneural function.

Sympathoadrenal imbalance could contribute to decreases in FVR and SVR via unopposed beta-2 adrenoceptor-mediated skeletal vasodilation driven by EPI (Goldstein et al., 2003), shunting cardiac output toward skeletal muscle and away from the brain (Supplementary Figure S3). High circulating EPI would also explain pallor, diaphoresis, and piloerection in NMH, via alpha-adrenoceptor agonism at cutaneous blood vessels, eccrine sweat glands, and arrector pili muscles. Studies have documented increases in plasma EPI levels *before* the onset of hypotension or presyncope (Robinson and Johnson, 1988; Benditt et al., 2003; Jardine et al., 1997). Serial blood sampling for plasma catecholamines has identified sympathoadrenal imbalance before NMH (Goldstein et al., 2003).

The finding of higher mean LF_RR_ *before* tilting in the NMH group raises the possibility that repetitive cues related to previous episodes of syncope might become classically conditioned stimuli (Norcliffe-Kaufmann et al., 2022). Animal models provide limited evidence that classical conditioning alters baroreflex function (Dworkin and Dworkin, 1995; Shammas et al., 1988; Hatton et al., 1984; Fitzgerald et al., 1984). We previously reported that across a variety of autonomic conditions and individual subjects, the log of LF_RR_ during supine rest is strongly positively correlated with the log of baroreflex-cardiovagal gain (Moak et al., 2007; Rahman et al., 2011; Goldstein et al., 2011). Thus, empirical clinical data relate supine LF_RR_ to baroreflex-cardiovagal function, although LF_RR_ may not itself provide a direct, specific measure of baroreflex-cardiovagal gain. In the present study, the rapid, precipitous decrease in blood pressure at the time of NMH without corresponding reflex tachycardia indicates abrupt withdrawal of baroreflex-cardiovagal function. The concurrent marked fall in LF_RR_ is consistent with the previously demonstrated relationship between LF_RR_ and baroreflex-cardiovagal function. However, the higher mean LF_RR_ during supine rest in the group that subsequently developed NMH could not be attributed to greater baseline baroreflex-cardiovagal gain, because none of the directly measured indices of baroreflex-cardiovagal function differed between the groups. The unexpected dissociation between elevated baseline LF_RR_ and the directly measured indices of baroreflex-cardiovagal function should be evaluated prospectively.

### Limitations

The small sample size is a limitation. Because of the small number of participants, there was a risk of false-negative results; however, in our view, the several strongly positive statistical results cast aside the null hypothesis that the results can be attributed to chance. FBF measurements can be substantially affected by artifact as NMH develops. Although none of the subjects had autonomic failure and the physiological effects of a Valsalva maneuver are expected to be short-lived, lasting less than 1 min before the blood pressure and heart rate return to baseline, we cannot rule out the possibility that the maneuver influenced baseline hemodynamics and catecholamine measurements. Varying tilt durations across subjects may have influenced neurohumoral responses and, therefore, the comparability of results. Specifically, a longer tilt duration allows secondary compensatory vasoconstriction through progressive baroreflex and myogenic adjustments; time-dependent reductions in venous return force progressive shifts in ventricular filling and ejection volumes; and prolonged sympathetic activation influences sympathoadrenal balance and heart rate variability indices. However, the median time to NMH was 15.1 min, close to the 20-min mark for measurements in the control group, and the groups did not differ in any variables at T1. Furthermore, we matched the analysis window (3 min before NMH and 3 min before 20 min of tilt). Therefore, we believe that unequal tilt durations had minimal influence on the results. Heterogeneity within NMH and No NMH groups, including a wide range of underlying conditions and concomitant medications, may have influenced the results but cannot explain the large number of statistically significant findings. While reflecting “true” autonomic tone, we acknowledge that unpaced breathing may have influenced heart rate variability results by showing higher LF_RR_ and greater data variability due to natural fluctuations in respiratory rate. High LF_RR_ during supine rest in patients with subsequent NMH was unexpected and should be confirmed in another cohort. Finally, the lack of MSNA measurement limits investigation of baroreflex sympathoneural function.

## Conclusion

The present results fit with the concept that a tilt-induced fall in central blood volume can trigger a neuroendocrine pattern that includes sympathoadrenal imbalance, resulting in acute decreases in skeletal vasomotor tone, CO, SVR, and shunting of the cardiac output away from the brain, which would be expected to reduce cerebral perfusion. In a distressing situation where a person can neither fight nor flee, a fainting reaction, by giving the impression of sudden death, might be lifesaving.

## Data Availability

The raw data supporting the conclusions of this article will be made available by the authors, without undue reservation.
