# Supplementary material for: Hemodynamics, plasma catecholamines, and power spectral analysis of heart rate variability during tilt-evoked neurally mediated hypotension
Source: Front Neurosci. 2026 Oct 6;20:1929006. doi: 10.3389/fnins.2026.1929006 (PMC13684423; doi:10.3389/fnins.2026.1929006)
Supplement: Supplementary file 1 [file Table_1.docx]

**SUPPLEMENTARY FILE**

**Details of tilt table testing and instrumentation**

Each subject reported to a dedicated autonomic function testing room at the NIH Clinical Center. The time of day of the testing was not controlled. With the subject supine an intravenous catheter (IV) was inserted into an arm vein and connected by a 3-way stopcock to an ongoing normal saline infusion at a slow rate to keep the vein open. Non-invasive monitoring devices included electrocardiographic leads, a brachial automated cuff and mercury in silastic strain gauge for measurement of forearm blood flow (FBF) by impedance plethysmography [3], a laser-Doppler flowmeter on the pad of the thumb for monitoring cutaneous microcirculatory flux, metal plates on two fingers for tracking skin electrical conductance (SEC, a measure of sweating), a thermistor for finger temperature, a thoracic circumferential device for measuring respiratory excursion by impedance plethysmography, a brachial cuff for manual blood pressure measurement, and a finger cuff for continuous tracking of blood pressure (Nexfin system, BMEYE, Amsterdam, the Netherlands; or Finapres Nova system, Finapres Medical Systems, Enschede, The Netherlands).

Blood pressure was monitored both by the automated finger cuff device and intermittently by the brachial cuff placed on the opposite arm. Cardiac stroke volume (SV) in mL was calculated and displayed from software bundled with the Nexfin and Nova systems. Forearm blood flow (FBF) was measured by impedance plethysmography.

Prior to tilting there was a period of at least 3 minutes with the subject resting supine to obtain baseline heart rate variability data. Then each subject performed a Valsalva maneuver (30 mmHg, 12 seconds) at least 3 times until a technically adequate tracing was obtained [2]. A blood sample was drawn through the indwelling IV for plasma catechols. FBF was measured 6 times by inflating the brachial cuff to 40 mmHg (above venous pressure, below diastolic pressure). Velcro straps were then applied around the legs and upper abdomen in preparation for tilting. A sock was removed, to enable visualization of dependent rubor.

For head-up tilt table testing, the motorized tilt table was brought to 70 degrees from horizontal over about 5-10 seconds. Beginning at about 2.5 minutes of tilting, FBF was measured, and at 4 minutes a blood sample was drawn through the IV. The patient and physiological variables were monitored continuously by at least two investigators, one viewing the signals and one the patient. FBF and blood sampling were repeated every 4 minutes for up to 40 minutes and after 10 minutes of supine recovery. There was no attempt to provoke hypotension by a drug. No subject lost consciousness.

Baroreflex-cardiovagal function was quantified from the slope of the linear relationship between cardiac interbeat interval (in seconds) and the systolic blood pressure (in mmHg) during Phase II of the Valsalva maneuver, as described previously [4]. To evaluate baroreflex-sympathoneural function we used the baroreflex areas method for Phases II and III/IV of the Valsalva maneuver [6].

Heart rate variability in the time and frequency domains was assessed using the HRV module bundled with the PowerLab recording device (LabChart 8.1.13, ADInstruments, Colorado Springs, CO). Low-frequency power of heart rate variability (LFRR) in msec^2^ and high-frequency power (HFRR) in msec^2^ were measured from the continuously recorded electrocardiogram for a period of at least 3 minutes during the monitoring period before the Valsalva maneuver. Window length for HRV was between 3 and 4 minutes for all timepoints. The log of LFRR has been proposed as an index of the ability to modulate cardiac autonomic outflows via baroreflexes [1]. The HFRR component (0.15 Hz to 0.4 Hz) provides an index of respiratory sinus arrhythmia, which depends on vagal efferent activity; the ratio of LFRR/HFRR , has been proposed to be an index of sympathovagal balance [5].

Cardiac output (CO) was calculated from the SV times the simultaneously measured heart rate (HR). FBF was in units of mL/min/100 mL, and the forearm vascular resistance (FVR) was calculated from the mean arterial pressure (MAP) divided by the FBF. Systemic vascular resistance (SVR, in mmHg·s/mL) was calculated from the MAP (-6 mmHg for assumed central venous pressure), the quantity divided by the CO.

Microcirculatory flux measured by laser-Doppler flowmetry (thumb) and finger temperature (thermistor) were recorded but not analyzed due to artifacts in most participants, which rendered the analysis invalid.

**SUPPLEMENTARY TABLES**

**Table S1. Subject characteristics**

|  | **Tilt Evoked Neurally-mediated hypotension (NMH)** | **No NMH** | **p-value** |
| --- | --- | --- | --- |
| N | 25 | 34 |  |
| Gender (female/male) | 13/12 | 13/21 | 0.4 |
| Age (years), mean (SD) | 40.3 (3.0) | 45.5 (2.6) | 0.2 |
| Body mass (kg), mean (SD) | 76.0 (3.4) | 75.7 (2.6) | 0.9 |
| Time to tilt down (median, min) | 15.1 | 40 | <0.001 |
| Referral diagnosis |  |  |  |
| Healthy volunteer | 8 | 14 |  |
| Parkinson disease | 1 | 0 |  |
| ME/CFS | 8 | 14 |  |
| PASC | 0 | 2 |  |
| Hypertryptasemia | 1 | 0 |  |
| POTS | 4 | 0 |  |
| Gulf War Illness | 3 | 4 |  |
| History of syncope (N) | 7/25 | 3/34 | 0.08 |
| History of orthostatic intolerance (N) | 17/25 | 11/34 | 0.01 |
| Medications |  |  |  |
| No medication (N) | 13 | 21 |  |
| Fludrocortisone (N) | 2 | 0 |  |
| Midodrine (N) | 2 | 0 |  |
| Pyridostigmine (N) | 1 | 1 |  |
| SSRI (N) | 4 | 6 |  |
| Benzodiazepine (N) | 2 | 1 |  |
| Antiepileptic (non benzodiazepine) (N) | 2 | 3 |  |
| Amphetamine (N) | 2 | 1 |  |
| ACE inhibitors (N) | 1 | 2 |  |
| ARBs (N) | 1 | 1 |  |
| HCTZ (N) | 2 | 1 |  |
| Alpha-adrenoceptor blocker (N) | 0 | 2 |  |
| Beta-adrenoceptor blocker (N) | 2 | 4 |  |
| Antibiotic / antiviral (N) | 1 | 3 |  |
| Supplements / vitamins (N) | 4 | 9 |  |
| NSAIDs (N) | 3 | 4 |  |
| Opioids (N) | 0 | 1 |  |
| Antihistamine (N) | 2 | 5 |  |
| Antiemetic (N) | 1 | 0 |  |
| Hormonal treatment (N) | 4 | 5 |  |
| Opiate antagonist (N) | 1 | 2 |  |
| GLP1-agonist | 0 | 1 |  |

**Abbreviations:** ACE inhibitors: angiotensin-converting enzyme inhibitors; ARBs: angiotensin II receptor blockers; HCTZ: hydrochlorothiazide; GLP1: glucagon-like peptide-1; ME/CFS: Myalgic Encephalomyelitis/Chronic Fatigue Syndrome; NSAIDs: nonsteroidal anti-inflammatory drugs; PASC: Post-Acute Sequelae of COVID-19; SSRI: selective serotonin reuptake inhibitors. Orthostatic intolerance was defined by a history of dizziness, lightheadedness, or weakness while standing, based on medical chart review.

**Table S2. Hemodynamic parameters, skin electrical conductance, and respiratory rate during supine rest (T0), at the start of the head-up tilting (T1), during tilt-evoked neurally mediated hypotension (NMH) or after 20 minutes of asymptomatic head-up tilt for controls (T2), and during supine recovery (T3).**

| **Condition** | **BPs**  *mmHg*  Mean (SEM) | **MAP**  *mmHg*  Mean (SEM) | **SV**  *mL*  Mean (SEM) | **HR**  *bpm*  Mean (SEM) | **CO**  *L/min*  Mean (SEM) | **FBF**  *cc/dl/min*  Mean (SEM) | **FVR**  *units*  Mean (SEM) | **SVR**  *units*  Mean (SEM) | **RR**  *per min*  Mean (SEM) |
| --- | --- | --- | --- | --- | --- | --- | --- | --- | --- |
| **Tilt-Evoked Neurally Mediated Hypotension (NMH)** | | | | | | | | | |
| **T0** | 132 (2.8) | 93 (2.1) | 84 (5.6) | 65 (2.1) | 5.5 (0.4) | 4.8 (1.7) | 50 (6.3) | 18 (1.6) | 17 (0.7) |
| **T1** | 128 (3.3) | 93 (2.4) | 59 (4.3) | 84 (4.3) | 4.8 (0.4) | 4.8 (1.5) | 46 (6.1) | 21 (2.4) | 15 (0.6) |
| **T2** | 86 (3.3), | 64 (3.3) | 52 (3.9) | 78 (5.7) | 4.1 (0.4) | 6.4 (1.5) | 23 (3.6) | 16 (1.8) | 18 (1.4) |
| **T3** | 131 (3.0) | 94 (2.5) | 79 (4.9) | 65 (2.2) | 5.3 (0.3) | 4.3 (1.4) | 51 (6.2) | 18 (1.5) | 16 (0.9) |
| **No NMH** | | | | | | | | | |
| **T0** | 125 (2.5) | 88 (1.5) | 94 (3.7) | 66 (1.7) | 6.1 (0.2) | 2.9 (0.3) | 41 (3.3) | 14 (0.5) | 18 (0.7) |
| **T1** | 130 (2.9) | 96 (2.1) | 69 (2.8) | 79 (2.1) | 5.3 (0.2) | 2.9 (0.2) | 43 (3.2) | 18 (0.8) | 16 (0.5) |
| **T2** | 128 (3.4) | 96 (2.3) | 62 (2.5) | 82 (2.5) | 5.1 (0.2) | 2.9 (0.2) | 41 (3.0) | 19 (0.9) | 17 (0.4) |
| **T3** | 124 (3.0) | 91 (2.1) | 85 (3.6) | 66 (1.5) | 5.5 (0.2) | 2.6 (0.3) | 49 (4.0) | 16 (0.7) | 17 (0.6) |

**Abbreviations:** BPs=systolic blood pressure; MAP=mean arterial pressure; SV=stroke volume; HR=heart rate; CO=cardiac output; FBF=forearm blood flow; FVR=forearm vascular resistance; SVR=systemic vascular resistance; RR=respiratory rate.

**Table S3. Heart rate variability and plasma catecholamine concentrations during supine rest (T0), at the start of the head-up tilting (T1), during tilt-evoked neurally mediated hypotension (NMH) or after 20 minutes of asymptomatic head-up tilt for controls (T2), and during supine recovery (T3).**

| **Condition** | **LFRR**  *msec^2^*  Mean (SEM) | **HFRR**  *msec^2^*  Mean (SEM) | **NE**  *nmol/L*  Mean (SEM) | **EPI**  *nmol/L*  Mean (SEM) |
| --- | --- | --- | --- | --- |
| **NMH** | | | | |
| **T0** | 1186 (153) | 622 (107) | 2.0 (0.2) | 0.12 (0.02) |
| **T1** | 613 (105) | 322 (114) | 3.2 (0.3) | 0.18 (0.03) |
| **T2** | 326 (80) | 126 (34) | 3.3 (0.3) | 0.48 (0.09) |
| **T3** | 814 (134) | 652 (112) | 1.9 (0.2) | 0.15 (0.02) |
| **No NMH** | | | | |
| **T0** | 683 (96) | 553 (168) | 1.7 (0.2) | 0.11 (0.02) |
| **T1** | 583 (135) | 203 (47) | 2.5 (0.2) | 0.17 (0.02) |
| **T2** | 506 (98) | 150 (30) | 2.9 (0.2) | 0.27 (0.04) |
| **T3** | 911 (181) | 800 (245) | 1.8 (0.3) | 0.13 (0.03) |

**Abbreviations:** LFRR=low frequency power of heart rate variability; HFRR=high frequency power of heart rate variability; NE=plasma norepinephrine; EPI=plasma epinephrine

**Table S4.** Effects of study periods (T0, T1, T2, T3) and groups (NMH vs. no NMH) on values for hemodynamic and neurochemical parameters using an analysis of variance (ANOVA)^#^ or mixed model effect^!^

|  | **Sum of Squares** | **Degrees of Freedom** | **Mean Square** | **F value** | **P value** |
| --- | --- | --- | --- | --- | --- |
| **BPs**^#^ | | | | | |
| Period x Group | 23597 | 3 | 7866 | (3, 171) = 65.96 | <0.001 |
| Period | 20609 | 3 | 6870 | (2.536, 144.6) = 57.61 | <0.001 |
| Group | 3073 | 1 | 3073 | (1, 57) = 4.145 | 0.05 |
| **MAP**^#^ | | | | | |
| Period x Group | 11218 | 3 | 3739 | (3, 171) = 54.56 | <0.001 |
| Period | 6910 | 3 | 2303 | (2.392, 136.4) = 33.61 | <0.001 |
| Group | 2207 | 1 | 2207 | "F (1, 57) = 5.183" | 0.03 |
| **SV**^!^ | | | | | |
| Period x Group |  |  |  | (3, 159) = 0.6660 | 0.57 |
| Period |  |  |  | (2.342, 124.1) = 74.39 | <0.001 |
| Group |  |  |  | (1, 54) = 4.467 | 0.04 |
| **HR**^#^ | | | | | |
| Period x Group | 758.2 | 3 | 252.7 | (3, 171) = 2.282 | 0.08 |
| Period | 13376 | 3 | 4459 | (1.633, 93.08) = 40.25 | <0.001 |
| Group | 1.405 | 1 | 1.405 | (1, 57) = 0.002296 | 0.96 |
| **CO**^!^ | | | | | |
| Period x Group | 4579680 | 3 | 1526560 | (3, 159) = 1.832 | 0.14 |
| Period | 43155537 | 3 | 14385179 | (2.237, 118.5) = 17.26 | <0.001 |
| Group | 17097756 | 1 | 17097756 | (1, 53) = 2.868 | 0.092 |
| **FBF**^!^ | | | | | |
| Period x Group |  |  |  | (3, 157) = 3.848 | 0.01 |
| Period |  |  |  | (1.619, 84.74) = 5.387 | 0.01 |
| Group |  |  |  | (1, 56) = 3.476 | 0.070 |
| **FVR**^!^ | | | | | |
| Period x Group |  |  |  | F (3, 155) = 9.131 | <0.001 |
| Period |  |  |  | (2.770, 143.1) = 14.00 | <0.001 |
| Group |  |  |  | (1, 56) = 0.1248 | 0.73 |
| **SVR**^!^ | | | | | |
| Period x Group |  |  |  | (3, 158) = 6.614 | <0.001 |
| Period |  |  |  | (2.117, 111.5) = 6.330 | 0.002 |
| Group |  |  |  | (1, 53) = 1.266 | 0.27 |
| **RR**^#^ | | | | | |
| Period x Group | 56.80 | 3 | 18.93 | (3, 165) = 1.944 | 0.13 |
| Period | 104.0 | 3 | 34.65 | (1.970, 108.4) = 3.558 | 0.03 |
| Group | 13.54 | 1 | 13.54 | (1, 55) = 0.4496 | 0.51 |
| **Log LFRR**^!^ | | | | | |
| Period x Group |  |  |  | (3, 170) = 7.387 | <0.001 |
| Period |  |  |  | (2.292, 129.9) = 23.53 | <0.001 |
| Group |  |  |  | (1, 57) = 0.07909 | 0.78 |
| **Log HFRR**^!^ | | | | | |
| Period x Group |  |  |  | F (3, 170) = 3.084 | 0.03 |
| Period |  |  |  | (2.447, 138.7) = 62.49 | <0.001 |
| Group |  |  |  | (1, 57) = 0.0006352 | 0.98 |
| **NE**^!^ | | | | | |
| Period x Group |  |  |  | (3, 157) = 2.319 | 0.08 |
| Period |  |  |  | (2.051, 107.3) = 62.10 | <0.001 |
| Group |  |  |  | (1, 55) = 1.148 | 0.29 |
| **Epi**^!^ | | | | | |
| Period x Group |  |  |  | (3, 149) = 4.961 | 0.003 |
| Period |  |  |  | (1.275, 63.31) = 30.71 | <0.001 |
| Group |  |  |  | (1, 52) = 2.946 | 0.09 |
| **NE/Epi**^!^ | | | | | |
| Period x Group |  |  |  | (3, 154) = 3.139 | 0.03 |
| Period |  |  |  | (1.579, 81.07) = 5.608 | 0.01 |
| Group |  |  |  | (1, 54) = 0.06656 | 0.807 |

**Abbreviations:** BPs=systolic blood pressure; MAP=mean arterial pressure; SV=stroke volume; HR=heart rate; CO=cardiac output; FBF=forearm blood flow; FVR=forearm vascular resistance; SVR=systemic vascular resistance; RR=respiratory rate. LFRR=low frequency power of heart rate variability; HFRR=high frequency power of heart rate variability; NE=plasma norepinephrine; EPI=plasma epinephrine.

**Table S5. Measures of baroreflex function in subject groups with or without tilt-evoked neurally mediated hypotension (NMH).**

| **Group** | **BRA-II**  *mmHg-s*  Mean (SEM) | **BRA-IV**  *mmHg-s*  Mean (SEM) | **Total area**  *mmHg-s*  Mean (SEM) | **PRT**  *s*  Mean (SEM) | **Baro slope**  *ms/mmHg*  Mean (SEM) |
| --- | --- | --- | --- | --- | --- |
| **NMH** | 107 (17.4) | 41 (5.0) | 151 (21.8) | 2.8 (0.2) | 4.6 (0.6) |
| **Controls** | 97 (17.7) | 66 (14.4) | 150 (29.0) | 3.1 (0.4) | 5.3 (0.5) |

There were no significant differences in baroreflex measures between the two groups. We used an independent-samples t-test.

**Abbreviations:** BRA-II=area under the curve (baroreflex area, BRA) between baseline systolic blood pressure and the BPs for each beat in Phase II; BRA-IV=area under the curve between baseline systolic blood pressure and the BPs for each beat in Phase IV; PRT=pressure recovery time (time interval from the bottom of phase III to complete return of BPs to baseline); Baro slope=slope of the linear regression line of the change in RR interval in msec per mm Hg change in BPs.

**SUPPLMENTARY FIGURE LEGENDS**

**Figure S1.** **Individual tracings of heart rate (HR), blood pressure (BP), and respiration in a representative subject with tilt-evoked neurally mediated hypotension (NMH) and a representative subject without NMH during supine rest, the asymptomatic phase of 70° head-up tilt (Tilt Start), tilt-evoked neurally mediated hypotension, and supine recovery.** Note the BP oscillation after the start of the 70 ° head-up tilt and the progressive hypotension without appropriate compensatory increase in heart rate. There is no significant change in respiratory rate or amplitude during the head-up tilt table test.

**Figure S2. Individual values for low frequency power of heart rate variability (LFRR) during supine rest (T0), at the start of the head-up tilt test (T1), during tilt-evoked neurally mediated hypotension (NMH) or after 20 minutes of asymptomatic head-up tilt position in subjects without NMH (T2), and during supine recovery (T3).** There was a significant decrease in LFRR from supine rest to T1 and from T1 to T2 in the group with NMH.

**Figure S3 – Concept diagram for the development of tilt-evoked neurally mediated hypotension (NMH)** The diagram (supplementary Figure) depicts NMH developing in three stages.

(Panel A) The first stage is baroreflexive negative feedback regulation (homeostasis). Green (+) signs indicate positive and (-) signs negative influences. In a negative feedback loop there is an odd number of (-) signs. For instance, with head-up tilting there is a decrease in cardiac filling (negtive #1), which in the absence of the baroreflex would decrease blood pressure; however, the decrease in cardiac filling inhibits firing of low pressure baroreceptors (negative #2), which restrain sympathetic noradrenergic system (SNS) outflows (negative #3). Release of the SNS from baroreceptor restraint increases blood pressure. There are 3 negative relationships in the loop. Blood pressure is maintained, because the increase in SNS outflow buffers the fall in blood pressure from the decrease in cardiac filling.

(Panel B) In the second stage is allostasis from involvement of higher centers. Based on factors such as learning (conditioning) and situational awareness, there is an increase in sympathetic adrenergic system (SAS) outflow, which via beta-2 adrenoceptors on vascular smooth muscle cells in skeletal muscle tends to counter-balance the reflexive SNS-mediated increase in skeletal muscle vascular resistance.

(Panel 3) NMH reflects a centrally-evoked neuroendocrine pattern that includes acceleration of SAS outflow while SNS outflow is shut down. This pattern involves multiple positive feedback loops, which are inherently unstable. For instance, because of the sudden failure of baroreflex-sympathoneural function and unopposed SAS effects on vascular resistances, the blood pressure plummets, and the patient manifests symptoms and signs of global cerebral hypoperfusion. The acceleration of SAS outflow evokes pallor, sweating, piloerection, trembling, and mydriasis, instinctively communicated signs of shock and distress.

**REFERENCES**

1.	Goldstein DS, Bentho O, Park MY, Sharabi Y (2011) Low-frequency power of heart rate variability is not a measure of cardiac sympathetic tone but may be a measure of modulation of cardiac autonomic outflows by baroreflexes. Exp. Physiol. 96:1255–1261

2.	Goldstein DS, Cheshire WP, Jr. (2017) Beat-to-beat blood pressure and heart rate responses to the Valsalva maneuver. Clin. Auton. Res. 27:361–367

3.	Goldstein DS, Holmes C, Frank SM, Naqibuddin M, Dendi R, Snader S, Calkins H (2003) Sympathoadrenal imbalance before neurocardiogenic syncope. Am. J. Cardiol. 91:53–58

4.	Goldstein DS, Horwitz D, Keiser HR (1982) Comparison of techniques for measuring baroreflex sensitivity in man. Circulation 66:432–439

5.	Guzzetti S, Piccaluga E, Casati R, Cerutti S, Lombardi F, Pagani M, Malliani A (1988) Sympathetic predominance in essential hypertension: a study employing spectral analysis of heart rate variability. J. Hypertens. 6:711–717

6.	Rahman F, Goldstein DS (2014) Quantitative indices of baroreflex-sympathoneural function. Clin. Auton. Res. 24:103–110
